# Hemorrhagic Pilocytic Astrocytomas in Adults: A Case Report and Literature Review

**DOI:** 10.7759/cureus.510

**Published:** 2016-02-24

**Authors:** Michael A Galgano, David J Padalino, Joseph Fullmer, Satish Krishnamurthy

**Affiliations:** 1 Neurosurgery, SUNY Upstate Medical University; 2 Crouse Neuroscience Institute, Crouse Medical Practice, PLLC; 3 Pathology, SUNY Upstate Medical University

**Keywords:** pilocytic astrocytoma, intratumoral hemorrhage, subdural hematoma, hemorrhagic tumor, hemorrhagic pilocytic astrocytoma, neurological compromise, suboccipital craniotomy, tumor resection

## Abstract

Pilocytic astrocytomas are histologically benign tumors, generally found in the pediatric population. Onset of symptoms is generally insidious, predominantly stemming from mass effect upon nearby structures. Patients harboring a pilocytic astrocytoma may present with gait disturbance, headaches, cranial nerve deficits, as well as hydrocephalus, depending on the exact location. Although cases of adult pilocytic astrocytomas in the adult population are described, they are quite uncommon. We present a case of an adult female presenting with acute neurological compromise resulting from an acutely hemorrhagic posterior fossa pilocytic astrocytoma. Her initial neurological assessment was consistent with a Glasgow coma scale of 4T, as the patient was experiencing decerebrate posturing. An emergent external ventricular drain was placed in the emergency department for acute hydrocephalus as a temporizing measure, prior to evacuation of the associated subdural and intratumoral hemorrhages, as well as resection of the mass. After a long hospital course and extensive rehabilitation, the patient made a remarkable recovery and eventually gave birth to a child via Caesarean section three years after her initial presentation.

## Introduction

Pilocytic astrocytomas are pathologically benign tumors typically seen in the pediatric population. Their clinical presentation is rather insidious from the onset and rarely acute. Depending on their location within the brain, and whether they are supratentorial or infratentorial, their symptomatology varies. Patients with supratentorial pilocytic astrocytomas may present with headaches and symptoms of a mass effect upon nearby structures, potentially leading to hydrocephalus or sensorimotor disturbances. Infratentorial pilocytic astrocytomas can also be a cause of hydrocephalus but may also cause compression of the cranial nerves with subsequent palsies.

In this paper, we aim to present an unusual case of an adult harboring a cerebellar pilocytic astrocytoma presenting in an acute fashion with a rapid neurological decline after spontaneous intratumoral hemorrhage. Reports in the literature of hemorrhagic presentation of these tumors exist, although they are quite rare [1-11]. The vast majority of pilocytic astrocytomas are not associated with hemorrhage, leading one to infer that a small subpopulation of these tumors may have inherent features making them more susceptible to hemorrhagic complications. Intratumoral encased aneurysms or dysplastic capillary beds may be contributing factors [5, 12]. In addition to this, some specific patient factors, such as older age, coagulation defects, and hypertension, may play a role [5, 12]. The case we are presenting highlights the importance of keeping pilocytic astrocytoma within the differential diagnosis in an otherwise healthy adult patient presenting with an unexplained intracranial hemorrhage.

## Case presentation

**Figure 1:**
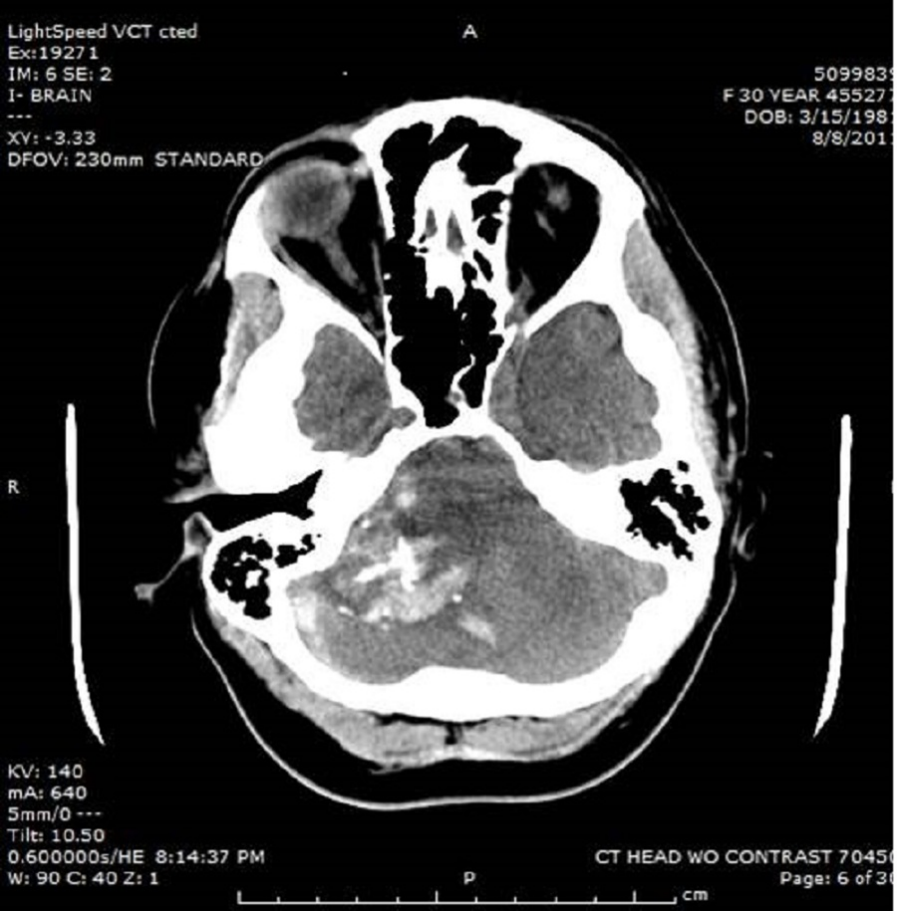
CT of the head revealing a right-sided cerebellar hemorrhagic calcified mass with effacement of the fourth ventricle.

A 30-year-old otherwise healthy female presented with the sudden onset of headache and subsequent obtundation. Upon arrival to our emergency department, her airway was secured with endotracheal intubation. Initial vital signs revealed bradycardia. The Glasgow coma scale score on admission was a 4T, as the patient had decerebrate posturing. Her eyes deviated downward with non-reactive, pinpoint pupils. Informed consent was waived due to the patient's neurological presentation. A CT scan of the head revealed a calcified lesion in the right cerebellar cortex with an acute intralesional hemorrhage and adjacent subdural hematoma. Effacement of the fourth ventricle with associated obstructive hydrocephalus was also noted (Figure 1).

**Figure 2:**
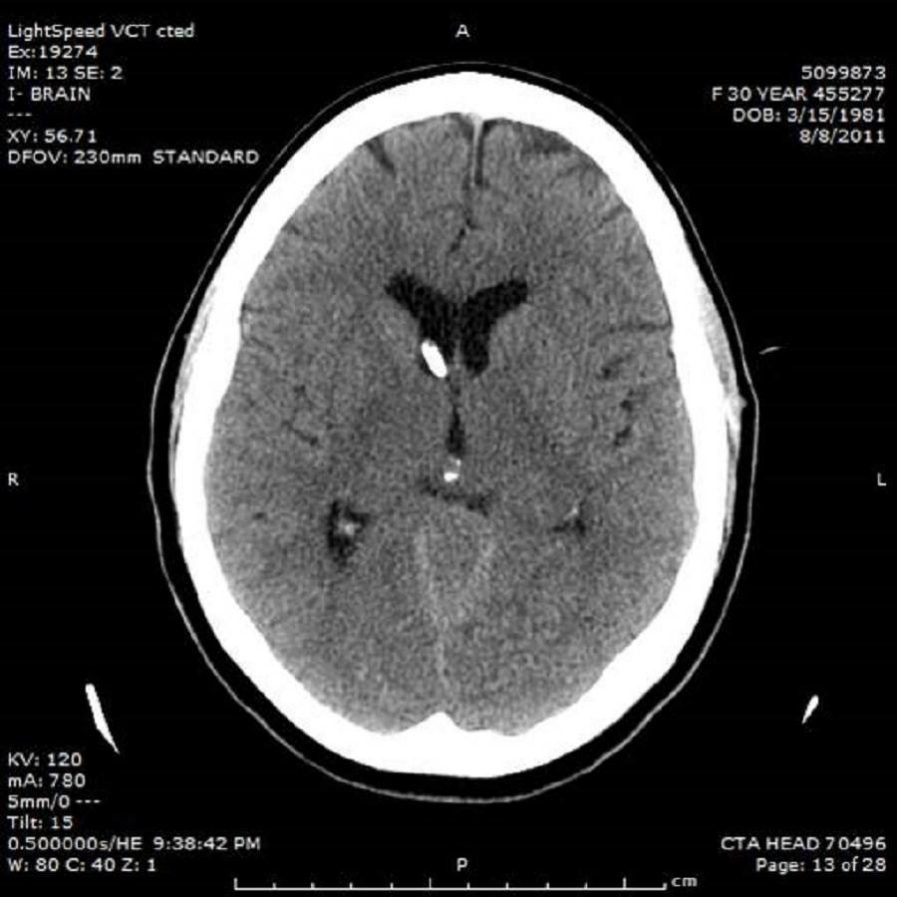
CT of the head revealing placement of a right frontal external ventricular drain for acute obstructive hydrocephalus.

An emergent right frontal external ventricular drain was placed in the emergency department as a temporizing measure. The opening pressure was over 30 cm H2O (Figure 2).

**Figure 3:**
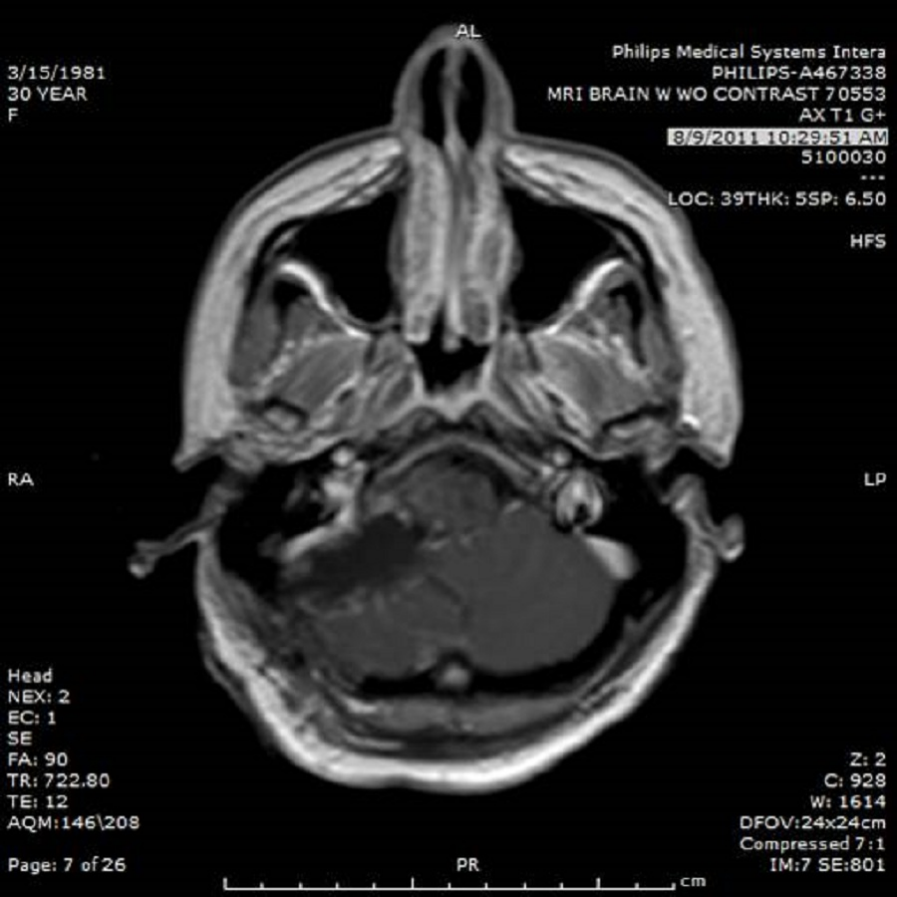
MRI of the brain with contrast detailing the resection cavity postoperatively.

A CT angiogram of the head and neck to rule out underlying vascular lesions was unremarkable. The patient was then taken to the operating room for surgical decompression. A paramedian suboccipital craniotomy was performed on the right side. The dura was opened and a subdural hematoma was quickly encountered and evacuated. An intra-axial cerebellar tumor rapidly presented as the dissection proceeded and a significant amount of intratumoral hemorrhage was noted. A gross total resection was undertaken (Figure 3).

**Figure 4:**
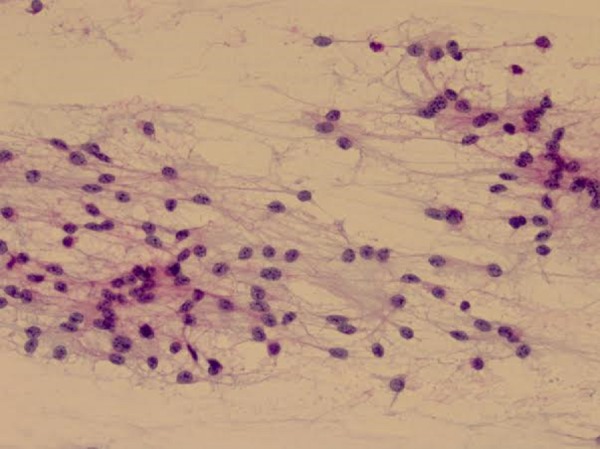
Smear at medium power. Relatively monomorphic cells with oval nuclei and bland cytology. Pilocytic processes are seen between the nuclei.

**Figure 5:**
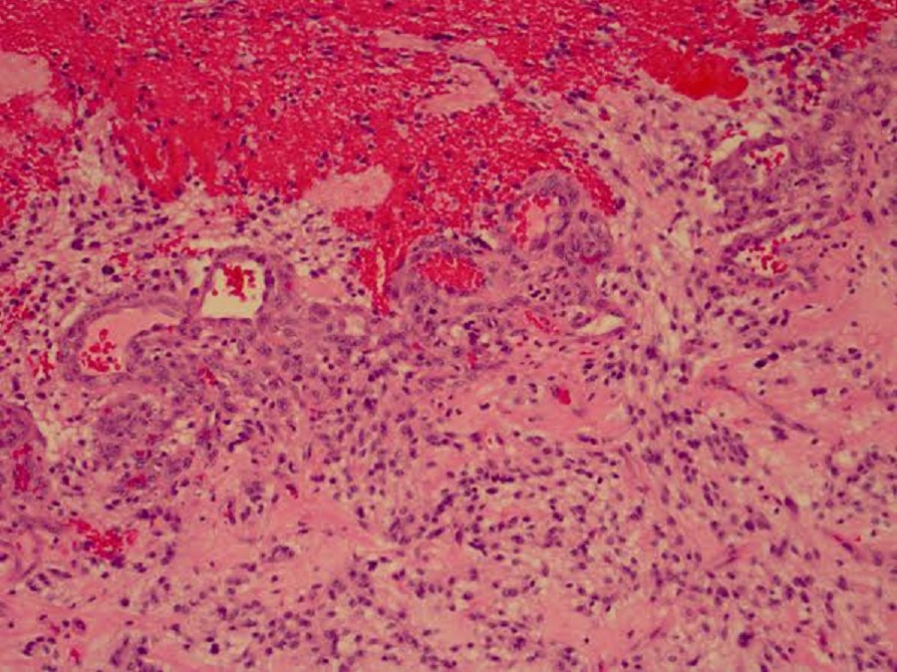
Pilocytic astrocytoma with endothelial proliferation and adjacent hemorrhage.

**Figure 6:**
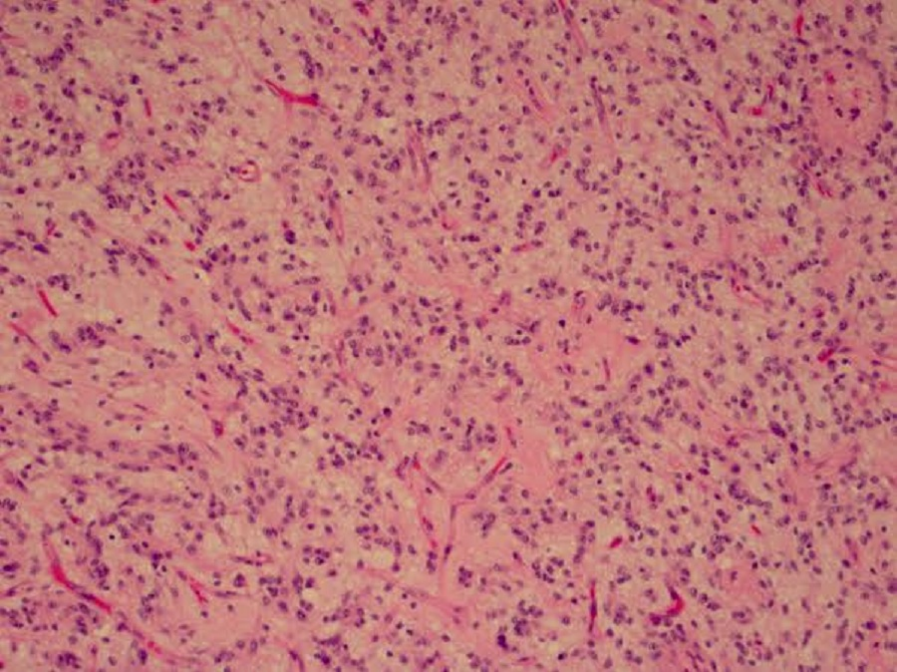
Pilocytic astrocytoma with prominent endothelial cells as well as neoplastic cells harboring mitotic figures and bland, oval nuclei.

A very small amount of tumor was purposefully left on the brainstem in an effort to avoid significant morbidity from an injury to the lower cranial nerves and the vertebral artery branches that were involved within the tumor. Final pathology of the tumor revealed a pilocytic astrocytoma (Figures 4-6).

The patient was following commands on the first postoperative day and was extubated a few days later. Postoperative neurological assessment revealed bilateral sixth cranial nerve palsies, right sensorineural hearing loss, and gait ataxia. The external ventricular drain was weaned and removed without any need for permanent CSF diversion. She was transferred for acute rehabilitation and was eventually discharged home with significant clinical improvement. At her one-year follow-up, she had minimal residual brainstem and cerebellar deficits. Adjuvant radiation therapy was considered, but the radiation oncologist felt that the risks of radiation therapy were not justified. She was able to walk independently and eventually return to work. Three years after her initial presentation, the patient got married and had a child via Caesarean section. During the postoperative period and during her pregnancy, her residual tumor has remained stable and no further episodes of hemorrhage or growth have been noted. Some of her symptoms, such as ataxia, got worse when she was pregnant but improved after delivery of the child.

## Discussion

Few reports exist describing adult pilocytic astrocytomas presenting in such an acute fashion with a cerebellar intratumoral hemorrhage, subdural hematoma, and acute obstructive hydrocephalus. Cases of hemorrhagic pilocytic astrocytomas have been reported in the literature, albeit on rare occasion. Golash, et al. reported on a case of a spontaneous intracerebral hemorrhage in a 13-year-old girl later found to be a pilocytic astrocytoma [2]. Lones and Verity described a fatal intracerebral hemorrhage in a 69-year-old woman caused by a pre-existing pilocytic astrocytoma [7]. Hwang, et al. described a case of a 34-year-old man presenting with a hemorrhagic hypothalamic pilocytic astrocytoma [3]. Oka, et al. reported on a 21-year-old man with a tectal pilocytic astrocytoma presenting with hemorrhage [9]. Lee, et al. described a case of a hemorrhagic cerebellar pilocytic astrocytoma in a 15-month-old boy [6]. Lyons reported on a case of a spontaneous intracerebral hemorrhage in association with a pilocytic astrocytoma in a 75-year-old man [8]. Recurrent pilocytic astrocytomas are rare; however, recurrence associated with an intracerebral hemorrhage has been noted in the literature [10]. Another rare presentation of pilocytic astrocytomas has been reported in association with subarachnoid hemorrhage [1, 4]. Perhaps the most comprehensive review of hemorrhage in association with pilocytic astrocytomas was completed by White and colleagues. One hundred and thirty-eight patients with histologically proven pilocytic astrocytomas were evaluated. The mean age at the time of diagnosis was 23 years. Approximately 8% of these individuals were found to have some degree of hemorrhage during their presentation. Although this number may seem higher than one would expect, their analysis found none of the hemorrhages to be in the cerebellum [11].

The pathophysiology leading to intratumoral hemorrhage of a pilocytic astrocytoma remains unclear. Higher grade astrocytomas present with hemorrhage more commonly as a result of vessel necrosis, rapidity of tumor cell proliferation, or secondary to neovascularization. Some contributing factors that are speculated in the literature to the etiology of hemorrhagic pilocytic astrocytomas include preexisting hypertension, abundant neovascularization of the tumor, structural abnormalities from tumor cell invasion of the vasculature, coagulation defects, endothelial proliferation, rupture of encased aneurysms, and dysplastic capillary beds [5, 12]. Another theory is increased fibrinolytic activity secondary to the thromboplastin activity of brain tissue. When a pilocytic astrocytoma does hemorrhage, the vast majority of it is intratumoral. When subdural hematoma and subarachnoid hemorrhage are present, this is typically accounted for by direct extension of the intratumoral hemorrhage into other spaces [5, 12].

The tumor itself can also grow into the subdural space, causing traction on communicating veins. This traction can cause the vessels to become more susceptible to rupture, even from an otherwise minor trauma [3]. This may lead to associated subdural hemorrhage. Shibao, et al. reported a case of a hemorrhagic pilocytic astrocytoma in an adult. They found that there was complex vascular proliferation within the tumor. This could potentially be accounted for by previously ruptured intratumoral vessels with subsequent recanalized thrombi. It was also noted that some of the vasculature included thin-walled ectatic vessels while other areas displayed sclerotic thick-walled vessels. Rupture within areas of this abnormal vasculature may be the etiology for intratumoral hemorrhage of pilocytic astrocytomas. It has also been implied that some degree of degenerative vascular changes may be the underlying factor ultimately leading to hemorrhage, given the older age distribution of patients with hemorrhagic pilocytic astrocytomas [9].

## Conclusions

Our patient presented with several features, making this a unique case. The patient described in this paper made remarkable improvements during her rehabilitation. Three years after her surgery, she delivered a child via Caesarean section and had no increase in the size of the small amount of residual tumor during her pregnancy. She has not had any more hemorrhage into the tumor. Underlying pilocytic astrocytomas should be considered in the differential diagnosis of adults presenting with an unexplained cerebellar hemorrhage.
